# The effect of whole grain wheat sourdough bread consumption on serum lipids in healthy normoglycemic/normoinsulinemic and hyperglycemic/hyperinsulinemic adults depends on presence of the *APOE *E3/E3 genotype: a randomized controlled trial

**DOI:** 10.1186/1743-7075-7-37

**Published:** 2010-05-05

**Authors:** Amy J Tucker, Kathryn A MacKay, Lindsay E Robinson, Terry E Graham, Marica Bakovic, Alison M Duncan

**Affiliations:** 1 Department of Human Health and Nutritional Sciences, University of Guelph, Guelph, Ontario, Canada, N1G 2W1

## Abstract

**Background:**

Epidemiological studies associate consumption of whole grain foods, including breads, with reduced cardiovascular disease (CVD) risk; however, few studies have compared wheat whole grains with wheat refined grains.

**Methods:**

This study investigated effects of 6-week consumption of whole grain wheat sourdough bread in comparison to white bread on fasting serum lipids in normoglycemic/normoinsulinemic (NGI; n = 14) and hyperglycemic/hyperinsulinemic (HGI; n = 14) adults. The influence of single-nucleotide polymorphisms, 3 within the *APOE *gene (E2, E3, E4) and 2 within the hepatic lipase gene promoter (*LIPC *-514C>T, LIPC -250G>A) were considered.

**Results:**

At baseline, HGI participants had significantly higher body weight, waist circumference, body fat, and fasted glucose, insulin, homeostasis model assessment of insulin resistance (HOMA-IR), glucagon, triacylglycerols (TAG) and TAG:HDL-cholesterol, compared to NGI participants; however, none of these in addition to none of the other serum lipids, differed between bread treatments, within either participant group. For participants with the *APOE *E3/E3 genotype, LDL-cholesterol (*P *= 0.02) increased in the NGI group (n = 7), and TAG (*P *= 0.03) and TAG:HDL-cholesterol (*P *= 0.04) increased in the HGI group (n = 10), following consumption of whole grain wheat sourdough compared to white bread.

**Conclusions:**

In summary, 6-week consumption of whole grain wheat sourdough bread did not significantly modulate serum lipids in NGI or HGI adults; however, it significantly increased LDL-cholesterol, TAG and TAG:HDL-cholesterol in participants with the *APOE *E3/E3 genotype. These data add to limited literature comparing wheat whole grains to wheat refined grains on CVD risk and highlight the need to consider genetic variation in relation to lipoprotein lipid content and CVD risk.

## Background

Epidemiological studies consistently associate consumption of whole grain foods, including whole grain breads, with reduced cardiovascular disease (CVD) risk [1-6] and CVD related co-morbidities [7-10]. Whole grains have the potential to reduce CVD risk through improvements in circulating lipids including decreased total-cholesterol, LDL-cholesterol and triacylglycerols (TAG), and increased HDL-cholesterol [11]. Although mechanisms for these effects are unclear, whole grain bioactive constituents such as dietary fiber, trace minerals, vitamins, lignans and phytochemicals may be potential contributors [12-14].

Research suggests that processing techniques that prepare the whole grain for digestion and absorption may not remove their bioactive constituents [15], therefore rationalizing the potential for breads made from whole grains to reduce CVD risk. Potential properties of whole grain breads such as sourdough fermentation [16] and large grain particle size [13] may provide additional health benefits including increased mineral bioavailability and improved postprandial glycemic response [17-20]. In contrast, refined grains found in white breads, contain more available carbohydrate and less dietary fiber, both of which may increase postprandial glycemia and circulating TAG leading to increased CVD risk [11,21].

Despite epidemiological evidence to support a role for whole grains in reducing CVD risk [1-6], results of intervention studies are inconsistent. Although intervention studies have replaced refined with whole grain foods [22-25], those that measured serum lipids did not attribute changes to the whole grains [22,24]. However, these investigations had study design limitations such as inclusion of whole grain foods produced from milled flour [22], use of a hypocaloric diet [24] and dependence on participants to self-select whole grain foods [24]. Further, although wheat was the dominant grain included [22-25], it is not known whether effects were due to wheat or non-wheat whole grains. Giacco et al. [26] addressed this by using only wheat products in a 3-week intervention of wholemeal wheat compared to refined wheat foods, which significantly decreased fasting total- and LDL-cholesterol in healthy adults. As well, other studies that have used less comprehensive interventions have demonstrated improved serum lipids following consumption of powdered wheat fibre [27]; high-protein, high-fibre wheat flakes [28]; and wheat whole grain and wheat bran cereal [29]. Potential mechanisms behind these effects include decreased glycemia, insulinemia and lipoprotein particles [30]. Studies that have focused on wheat breads have either not measured [31] or found no significant effect [32,33] on serum lipids, prompting the need for more whole grain wheat bread interventions.

In addition to the use of traditional CVD biomarkers to investigate the impact of whole grains, it is of value to consider individual genetic variation [34,35]. Single-nucleotide polymorphisms (SNPs), particularly the e missense mutations of the *APOE *gene [36] and those of the hepatic lipase gene promoter (*LIPC*) [37], are reflective of varying circulating lipid profiles and thus warrant study in relation to CVD risk assessment. In addition to dyslipidemia, these SNPs may also help identify additional isolated or combined CVD risk factors such as obesity and dysglycemia.

The purpose of the current study was to address the knowledge gap in whole grain wheat intervention studies by investigating effects of whole grain and refined wheat bread consumption on CVD risk while considering the influence of genetic variation. The primary objective of this dietary intervention was to determine the response to 6-week consumption of whole grain wheat sourdough bread in comparison to white bread, on fasted serum lipids and apolipoproteins, in healthy men and postmenopausal women of low and high levels of CVD risk. A secondary objective was to consider the influences of *APOE *and *LIPC *polymorphisms on the serum lipid responses to the bread treatments.

## Methods

### Participants

Men and postmenopausal women (defined as absence of menstruation ≥ 1 y) aged 43-70 y, were recruited using posters and newspaper advertisements. Participants were initially screened using an eligibility questionnaire and excluded if they reported the presence of any serious medical condition (i.e. CVD, diabetes, cancer, hepatic disease, renal disease), frequent alcohol consumption (≥ 2 drinks.d^-1^), use of lipid-lowering medication, use of hormone replacement therapy, use of daily aspirin, major body weight changes (> 5% of usual body weight within the last 6 months), current or planned attempts at trying to lose or gain weight, plans to alter their habitual lifestyle, elite athletic training or an allergy to gluten. Potential participants meeting these criteria then underwent an oral glucose tolerance test (OGTT) of 75 g dextrose (Trutol 75, NERL Diagnostics, RI, USA) to rule out diabetes (based on fasted and 2-h glucose) and to determine fasted and 2-h whole blood glucose and serum insulin. Potential participants were then considered eligible if they met the inclusion criteria of 1 of 2 groups representing different levels of CVD risk. Inclusion criteria for the normoglycemic/normoinsulinemic (NGI) group were: BMI 19-25 kg·m^-2 ^and/or waist circumference < 102 cm for men and < 88 cm for women, and all of the following: normal fasted glucose (plasma glucose < 6.1 mmol·L^-1^), normal glucose tolerance (2-h post-OGTT plasma glucose < 7.8 mmol·L^-1^), and normal fasted insulin (serum insulin < 90 pmol·L^-1^). Inclusion criteria for the hyperglycemic/hyperinsulinemic (HGI) group were: BMI > 29.9 kg·m^-2 ^and/or waist circumference ≥ 102 cm for men and ≥ 88 cm for women, and one of the following: fasted hyperglycemia (plasma glucose 6.1-6.9 mmol·L^-1^), impaired glucose tolerance (2-h post-OGTT plasma glucose 7.8-11.0 mmol·L^-1^), and/or fasted hyperinsulinemia (serum insulin > 90 pmol·L^-1^). A total of 28 men and postmenopausal women were included in the study (n = 14 NGI; 10 male, 4 female and n = 14 HGI; 10 male, 4 female). A sample size of 28 participants was estimated to be adequate for detection of a 10% change in fasting total-cholesterol using variation in accordance with the literature [22,24,26], an alpha of 0.05, a power of 0.80 and 2-sided testing. The study was approved by the Human Research Ethics Board of the University of Guelph and all participants provided written consent.

### Study design and treatment breads

The study utilized a randomized crossover design in which participants added 2 commercially available breads (whole grain wheat sourdough and white) to their habitual diets for 6 weeks each, separated by 4-5 week washout period. The whole grain wheat sourdough bread consisted predominantly of whole grain wheat flour (37% dry weight) and also contained other ingredients including non-wheat grains (18% dry weight: whole grain spelt and rye flours, brown flax seeds, rolled oats, cracked soy, yellow flax seeds, millet seeds, malted barley flour, brown rice flour, millet flour, durum semolina and organic sourdough made from natural bacterial culture, whole grain spelt and rye flours).

The quantity of treatment breads provided was determined to correspond with 65% of the daily grain serving recommendation of Canada's Food Guide (CFG) [38], which, based on a bread slice of 35 g, equated to 136.5-163.8 g bread·d^-1 ^(6-7 grain servings·d^-1^) for women and 159.3-182.0 g bread·d^-1 ^(7-8 grain servings/d) for men. The daily number of slices of treatment bread provided was then calculated using the average grain serving CFG recommendation based on bread weight divided by the weight of the treatment bread slices (25 g/slice for whole grain wheat sourdough bread and 37.5 g/slice for white bread). Therefore, the number of slices provided to women and men, respectively, was 6 and 7 for whole grain wheat sourdough bread and 4 and 5 for white bread. The nutritional composition of each daily amount of bread was then calculated accordingly (Table 1).

**Table 1:** Daily amount and composition of study treatment breads^1^

	Whole grain wheat sourdough bread		White bread	
	
	Male	Female	Male	Female
				
Number of slices	7	6	5	4
Total bread amount (g)	175.0	150.0	187.5	150.0
Energy (kJ)	1553	1331	1947	1557
Total carbohydrate (g)	64.8	55.5	85.5	68.4
Starch (g)	50.9	43.7	74.1	59.3
Total sugars (g)	4.90	4.20	10.9	8.70
Total dietary fiber (g)	14.9	12.8	8.44	6.75
Soluble fiber (g)	1.05	0.90	0.56	0.45
Insoluble fiber (g)	13.8	11.9	7.88	6.30
Protein (g)	19.6	16.8	16.7	13.4
Fat (g)	3.68	3.15	6.19	4.95

^1^Data were analyzed by Industrial Laboratories of Canada (Mississauga, Ontario, Canada).

Daily portions of the treatment breads were packaged into freezer bags and immediately distributed or frozen until distributed to participants in 2 week supplies on study d 1, 14 and 28. Participants were instructed to consume their treatment bread in place of other bread products (e.g. bagels, pitas, dinner rolls) or carbohydrate-rich foods (e.g. pasta, rice). Participants continued their habitual diet with the exception of avoiding natural health products (except for multi-vitamin/mineral supplements which were permitted).

### Data collection and sample analysis

Participants reported to the laboratory on d 0, 1, 14, 28, 42 and 43, of each treatment period. For each set of 3-d before a fasted blood sample, participants were instructed to avoid caffeine, alcohol and strenuous exercise. On d -1, 0, 41 and 42, participants were instructed to consume a standard meal by 8 pm consisting of a single-serving lasagna, apple or orange juice box, and chocolate or vanilla pudding or chocolate granola bar, after which, only water was permitted.

### Body measurements

Height was measured on d 0 using a standard measuring tape or a stadiometer (Seca Mod. 220, Germany) (depended on availability but consistent within a participant), with participants standing with their feet together and back and heels against the wall or the vertical bar of the stadiometer.

Body weight was measured on d 0, 1, 14, 28, 42 and 43 of each treatment period using an analog (Ohaus I-10, VA, USA), household (Hydro Scale, Taiwan) or balance beam (Seca Mod. 220, Germany) scale (depended on availability but consistent within a participant) with participants wearing a light shirt, no shoes and having empty pockets. BMI was calculated as body weight (kg)/height (m^2^).

Waist circumference was measured using a standard measuring tape on d 0, 1, 42 and 43 of each treatment period between the top of the iliac crest (hip bones) and the bottom of the rib cage with participants wearing a light shirt.

Body composition was measured on d 0, 1, 42 and 43 of each treatment period using bioelectrical impedance analysis (BIA) (BodyStat™ 1500, BodyStat Ltd., MI, USA) with participants resting in a supine position. Participants were instructed to consume at least 2 glasses of water the evening before their BIA measurement to increase the measurement's accuracy.

Diastolic and systolic blood pressure was measured in duplicate using a blood pressure monitor (Dinamap™ Plus Vital Signs Monitor, Johnson & Johnson Medical Inc., FL, USA) on d 0, 1, 42 and 43 of each treatment period, after the participants were rested for 5 min.

### Food records

Three-d food records were completed on d -3 to -1, 12 to 14, 30 to 32 and 39 to 41 of each treatment period. Participants were provided food record sheets with space to indicate time of food or drink consumption, amount and a detailed description. Food records were analyzed for 3-d average intakes of energy, protein, carbohydrate, dietary fiber, total fat, SFA, MUFA, PUFA and cholesterol using the ESHA Food Analysis program (Version 9.8.0, Salem, OR, USA).

### Glycemic parameters

Fasted venous blood samples were collected on d 0, 1, 42 and 43 of each treatment period for analysis of whole blood glucose, serum insulin and plasma glucagon. Data were averaged from d 0 and 1, and from d 42 and 43. To reduce the effects of inter-assay variability, all samples from the same participant were analyzed in the same assay.

Whole blood for glucose was prepared from blood collected into sodium heparin coated collection tubes (BD Vacutainer(tm), Becton, Dickinson and Company, NJ, USA), mixed thoroughly and kept on ice until measurement in duplicate using a glucose auto-analyzer (YSI2300 Stat Plus, YSI Inc., Yellow Springs, OH, USA). Intra-assay variability for glucose was 1.3%.

Serum for insulin was processed from blood collected into anti-coagulant-free collection tubes (Beckman Allegra^® ^Beckman Coulter Inc., CA, USA), left to clot at room temperature for 30 min, centrifuged at 1500 × g for 15 min at 5°C, aliquoted and frozen at -20°C until analysis. Serum insulin was analyzed using a single antibody-coated tube RIA with ^125^I-labeled hormone (Coat-A-Count, Siemens Medical Solution Diagnostics, CA, USA). Inter- and intra-assay variability for insulin was 1.4% and 1.9%, respectively.

Plasma for glucagon was prepared from blood collected into K_2 _EDTA collection tubes (BD Vacutainer™) with 6 μL of dipeptidyl peptidase IV inhibitor (Millipore, MO, USA). Within 1 min of collection, 90 μL of bovine lung aprotinin (Sigma Aldrich, MO, USA) was added and mixed thoroughly. Collection tubes were centrifuged 5-15 min later at 1000 × g for 10 min at 5°C and plasma was frozen at -20°C until analysis. Plasma glucagon was analyzed using a double antibody RIA with ^125^I-labeled hormone (Millipore, MO, USA). Inter- and intra-assay variability for glucagon was 3.7% and 3.6%, respectively.

The homeostasis model assessment of insulin resistance (HOMA-IR) was calculated as fasted whole blood glucose (mmol·L^-1^) × fasted serum insulin (pmol·L^-1^)/22.5.

### Serum lipids and apolipoproteins

Fasted venous blood samples were collected on d 0 and 43 of each treatment period for analysis of serum lipids and apolipoproteins into anti-coagulant-free collection tubes, left to clot at room temperature for 30 min, centrifuged at 1500 × g for 15 min at 5°C and frozen at -20°C until analysis. Serum total-cholesterol, HDL-cholesterol and TAG were measured at Guelph General Hospital (Guelph, ON, Canada) using an auto-analyzer (Synchron CX systems, Beckman Coulter, Mississauga, ON, Canada) with all samples from each participant in the same batch. LDL-cholesterol was calculated using the Friedewald equation when TAG ≤ 4.52 mmol·L^-1^) [39] and non-HDL-cholesterol was calculated as total-cholesterol minus HDL-cholesterol. Calculations were also performed for the ratios of total-cholesterol:HDL-cholesterol, LDL-cholesterol:HDL-cholesterol, TAG:HDL-cholesterol and non-HDL-cholesterol:HDL-cholesterol. Inter- and intra-assay variability, respectively, was 1.6% and 5.7% for total-cholesterol, 7.3% and 8.4% for HDL-cholesterol, and 8.2% and 4.8% for TAG.

Serum apolipoprotein B and apolipoprotein AI were analyzed at the Lipid Analytical Laboratory (Toronto, ON, Canada) by end-point nephelometry [40] using a Behring Nephelometer ProSpec(r) System Assay Loader (Dade Behring Inc., Mississauga, ON, Canada) with all samples from each participant in the same batch, and the apolipoprotein B/apolipoprotein AI ratio was calculated. Inter- and intra-assay variability, respectively, was 5.0% and 6.1% for apolipoprotein B, and 10.8% and 5.8% for apolipoprotein AI.

### Polymorphism genotyping

Venous whole blood for genotyping was collected into sodium heparin coated collection tubes, mixed thoroughly and refrigerated at 4°C for 1-2 d before analysis. Whole blood samples were collected on d 42 of the last treatment period for 14 of the participants and within 3 months of completion of the study for 13 of the participants (sample was not collected from 1 participant (male NGI)).

DNA was extracted from fresh heparinized whole blood using a QIAamp DNA Blood Mini Kit (Qiagen, CA, USA). Genomic DNA was then amplified using an Illustra GenomiPhi V2 DNA Amplification Kit (GE Healthcare, Baie d'Urfe, QC, Canada). The e missense SNPs of the *APOE *gene (E2, E3, E4) were characterized by methods described by Wood et al. [41]. Described briefly, following PCR amplification of the 244 bp fragment of the *APOE *gene, variants were identified by HhaI digestion, separated on an 18% polyacrylamide gel and visualized with SYBR green (Invitrogen, ON, Canada) staining. *LIPC *-514C>T genotyping was performed using methods described by Couture et al. [37]. Described briefly, following the amplification of the 285 bp fragment of the *LIPC *gene, variants were identified using NlaIII digestion, separated on a 6% polyacrylamide gel and visualized with SYBR green staining. *LIPC *-250G>A genotyping was performed using methods described by van't Hooft et al. [42]. Described briefly, following the PCR amplification of the 710 bp fragment of the *LIPC *gene promoter, variants were identified using DraI digestion, separated on a 1.5% agarose gel and visualized with ethidium bromide staining.

### Study diary

A daily study diary was kept during each treatment period in which participants were instructed to record how and when they consumed their treatment bread, physical activity, illness and medication use.

### Statistical analyses

The Statistical Analysis System, version 9.1 (SAS Institute, Cary, NC, USA) was used for all statistical analyses with significance set at *P *≤ 0.05 and unless indicated otherwise, all data are presented mean ± SEM. Examination of all data using box plots, stem leaf diagrams and normal probability plots revealed that serum insulin, plasma glucagon, HOMA-IR, serum TAG and TAG:HDL-cholesterol were not normally distributed and were therefore logarithmically transformed prior to statistical analyses and presented as geometric mean (95% CI).

Comparisons of participant baseline characteristics (body weight, BMI, body fat, waist circumference, blood pressure, whole blood glucose, serum insulin, HOMA-IR, plasma glucagon, serum lipids) between the NGI and HGI group were completed using unpaired Student's t-tests.

Comparison of serum lipids among identified genotypes (E2/E3, E3/E3 and E3/E4; -514CC and -514CT; -250GG and -250GA) within the NGI and HGI groups were completed using ANOVA, controlling for genotype, followed by the Tukey's test for multiple comparisons.

The effect of treatment on the average of d 42 and 43 values for body measurements (body weight, BMI, waist circumference, body fat) and glycemic parameters (fasted whole blood glucose, fasted serum insulin, HOMA-IR, fasted plasma glucagon) was determined within the NGI and HGI groups separately using repeated measures analysis of covariance (ANCOVA), controlling for participant, treatment period and treatment and including the average of d 0 and 1 values as a covariate, followed by the Tukey's test for multiple comparisons.

The effect of treatment on energy and nutrient (protein, carbohydrate, dietary fiber, total fat, SFA, MUFA, PUFA, cholesterol) intakes was determined within the NGI and HGI groups separately using unpaired Student's t-tests.

The effect of treatment on serum lipids at d 43 was determined within the NGI and HGI group separately using repeated measures ANCOVA, controlling for participant, treatment period and treatment and including d 1 values as a covariate, followed by the Tukey's test for multiple comparisons. The influence of genotype (E2/E3, E3/E3 and E3/E4; -514CC and -514CT; -250GG and -250GA) on the effect of treatment on serum lipids at d 43 was determined within the NGI and HGI group separately using repeated measures ANCOVA, controlling for participant, treatment period and treatment and including d 1 values as a covariate, followed by the Tukey's test for multiple comparisons.

## Results

### Participant characteristics

The 28 participants (n = 14 NGI, n = 14 HGI) who started the study completed both treatments with the exception of 1 female (HGI) who did not complete the whole grain wheat sourdough bread treatment due to personal reasons. Participants were Caucasian (n = 27) and African Canadian (n = 1, male HGI). At study entry, participants within the HGI group had significantly higher body weight, BMI, waist circumference, percent body fat, and fasted concentrations of whole blood glucose, serum insulin, HOMA-IR, plasma glucagon, serum TAG and serum TAG:HDL-cholesterol, when compared to participants within the NGI group (Table 2).

**Table 2:** Baseline characteristics of NGI and HGI adults^1, 2^

	NGI	HGI	P-value group effect^3^
n	14	14	
Gender (male (n)/female (n))	10/4	10/4	
Age (y)	53.1 ± 1.61	57.4 ± 1.97	0.10
Height (m)	1.73 ± 0.11	1.73 ± 0.09	0.97
Body weight (kg)	79.4 ± 3.66	107.4 ± 5.10	0.0001
BMI (kg·m^-2^)	26.5 ± 0.78	35.7 ± 1.51	< 0.0001
Waist circumference (cm)	93.4 ± 2.18	116.3 ± 2.96	< 0.0001
Body fat (%)	27.8 ± 2.03	36.0 ± 2.14	0.01
Systolic blood pressure (mmHg)^4^	116.9 ± 4.07	126.3 ± 3.16	0.08
Diastolic blood pressure (mmHg)^4^	66.0 ± 2.11	68.8 ± 2.60	0.45
Whole blood glucose (mmol·L^-1^)	4.47 ± 0.11	5.04 ± 0.10	0.0007
Serum insulin (pmol·L^-1^)^5^	30.9 (26.6, 35.8)	93.8 (80.7, 108.9)	< 0.0001
HOMA-IR^5^	0.85 (0.73, 0.98)	3.00 (2.60, 3.46)	< 0.0001
Plasma glucagon (ng·L^-1^)^5^	56.7 (48.1, 66.9)	71.0 (60.4, 83.5)	0.05
Serum total-chol (mmol·L^-1^)	5.10 ± 0.21	4.87 ± 0.26	0.50
Serum HDL-chol (mmol·L^-1^)	1.28 ± 0.10	1.06 ± 0.08	0.11
Serum LDL-chol (mmol·L^-1^)^5^	3.30 ± 0.18	2.98 ± 0.25	0.31
Serum TAG (mmol·L^-1^)^5^	1.06 (0.84, 1.34)	1.63 (1.22, 2.18)	0.02
Serum non-HDL-chol (mmol·L^-1^)	3.81 ± 0.21	3.81 ± 0.28	0.98
Total-chol:HDL-chol	4.26 ± 0.32	4.97 ± 0.46	0.22
LDL-chol:HDL-chol	2.82 ± 0.27	3.06 ± 0.32	0.57
TAG:HDL-chol^5^	0.87 (0.62, 1.21)	1.60 (1.09, 2.36)	0.01
Non-HDL-chol:HDL-chol	3.26 ± 0.32	3.97 ± 0.46	0.22
Apolipoprotein B (g·L^-1^)	0.92 ± 0.06	0.96 ± 0.07	0.65
Apolipoprotein AI (g·L^-1^)	1.54 ± 0.07	1.42 ± 0.06	0.21
Apolipoprotein B: Apolipoprotein AI	0.62 ± 0.05	0.70 ± 0.07	0.34

^1^Values are mean ± SEM unless otherwise indicated. Abbreviations: chol: cholesterol.

^2^All plasma and serum endpoints were collected in the fasted state.

^3^P-values for differences between NGI and HGI groups at study entry using ANOVA.

^4^Within the HGI group, n = 12 for whole grain wheat sourdough bread and n = 13 for white bread.

^5^Data were log-transformed prior to statistical analysis and presented as geometric mean (95% CI).

### Polymorphism genotyping

For the *APOE *polymorphisms, the identified proportions were 0.19 for the E2/E3 genotype (n = 2 NGI, n = 3 HGI); 0.63 for the E3/E3 genotype (n = 7 NGI, n = 10 HGI) and 0.19 for the E3/E4 genotype (n = 4 NGI, n = 1 HGI). There were no *APOE *E2/E2 or E4/E4 homozygous participants identified. For the *LIPC *polymorphisms (-514C>T and -250G>A), the identified participants and proportions were the same (due to complete linkage disequilibrium) at 0.74 for the common homozygous (CC and GG) genotypes (n = 8 NGI, n = 12 HGI) and 0.26 for the rare heterozygous (CT and GA) genotypes (n = 5 NGI, n = 2 HGI). There were no *LIPC *TT or AA homozygous participants identified. Additionally, there were no significant differences in any baseline serum lipids between the genotypes for either the *APOE *or *LIPC *polymorphisms (data not shown).

### Body measurements and glycemic parameters

Body measurements, including BMI, body fat, waist circumference and systolic and diastolic blood pressure, did not significantly differ at either day 1 or day 43 between the whole grain wheat sourdough and white bread treatments, within either the NGI or HGI group (data not shown). Body weight data also did not significantly differ between whole grain wheat sourdough and white bread treatments at either day 1 (NGI: 80.1 ± 3.64 kg and 79.4 ± 3.61 kg, respectively; HGI: 106.6 ± 5.85 kg and 106.9 ± 5.04 kg, respectively) or day 43 (NGI: 79.6 ± 3.61 kg and 79.3 ± 3.67 kg, respectively; HGI: 106.0 ± 5.71 kg and 106.4 ± 4.99 kg, respectively). Similarly, glycemic parameters, including fasted whole blood glucose, serum insulin, HOMA-IR and plasma glucagon, did not significantly differ at either day 1 or day 43 between the whole grain wheat sourdough and white bread treatments, within either the NGI or HGI group (data not shown).

### Energy and nutrient intakes

Daily intakes of energy, carbohydrate, protein, total fat, SFA, MUFA, PUFA or cholesterol did not significantly differ between the 3-d period before the start of each treatment, within either the NGI or HGI group (data not shown). During the study, intakes of dietary fiber (within the NGI and HGI group) and MUFA (within the NGI group) were significantly greater during the whole grain wheat sourdough compared to the white bread treatment (Table 3). There were no significant differences in the intakes of energy or other nutrients (protein, total carbohydrate, total fat, SFA, PUFA, cholesterol) between bread treatments, within either the NGI or HGI group (Table 3).

**Table 3:** Effect of 6-wk whole grain wheat sourdough bread consumption on energy and nutrient intakes in NGI and HGI adults^1^

	Whole grain wheat sourdough bread	White bread	P-value treatment effect^2^
n			
NGI	14	14	
HGI	13	14	
Energy (kJ)			
NGI	9726 ± 480	9986 ± 510	0.71
HGI	10140 ± 625	10634 ± 506	0.54
Protein (g)			
NGI	93.1 ± 5.18	87.9 ± 4.41	0.45
HGI	104.1 ± 8.66	108.0 ± 5.84	0.71
Total carbohydrate (g)			
NGI	291.9 ± 22.3	323.6 ± 23.0	0.33
HGI	284.2 ± 20.1	306.9 ± 18.7	0.41
Dietary fiber (g)			
NGI	31.2 ± 2.04	22.0 ± 1.88	0.003
HGI	29.6 ± 2.13	20.2 ± 1.62	0.002
Total fat (g)			
NGI	89.6 ± 5.00	83.9 ± 3.62	0.37
HGI	93.5 ± 7.40	95.5 ± 5.16	0.83
MUFA (g)			
NGI	20.4 ± 1.57	16.6 ± 0.99	0.05
HGI	25.5 ± 2.67	26.6 ± 2.48	0.75
Cholesterol (mg)			
NGI	228.7 ± 28.6	227.7 ± 15.6	0.98
HGI	326.5 ± 34.7	372.7 ± 31.7	0.33

^1^Values are mean ± SEM.

^2^P-value for treatment effect comparison of energy and nutrient intakes averaged over 3 sets of 3-d food records (d -3 to -1, 12 to 14, 30 to 32) within each treatment period using unpaired Student's t-test.

### Serum lipids and apolipoproteins

Fasted serum lipids, including total-cholesterol, HDL-cholesterol, LDL-cholesterol, TAG, apolipoprotein B and apolipoprotein AI, did not significantly differ between the whole grain wheat sourdough and white bread treatments, within either the NGI or HGI group (Table 4). Similarly, lipid ratios, including total-cholesterol:HDL-cholesterol, LDL-cholesterol:HDL-cholesterol, TAG:HDL-cholesterol, non-HDL-cholesterol:HDL-cholesterol and apolipoprotein B:apolipoprotein AI, did not significantly differ between the whole grain wheat sourdough and white bread treatments, within either the NGI or HGI group (data not shown).

**Table 4:** Effect of 6-wk whole grain wheat sourdough bread consumption on fasting serum lipids in NGI and HGI adults^1^

	Whole grain wheat sourdough bread		White bread		P-value treatment effect^2^
	
	Day 1	Day 43	Day 1	Day 43	
n					
NGI	14	14	14	14	
HGI	13	13	14	14	
Total-chol (mmol·L^-1^)					
NGI	5.04 ± 0.22	5.17 ± 0.18	5.19 ± 0.21	5.00 ± 0.19	0.06
HGI	4.77 ± 0.27	5.07 ± 0.29	4.96 ± 0.28	5.07 ± 0.31	0.71
HDL-chol (mmol·L^-1^)					
NGI	1.33 ± 0.13	1.33 ± 0.11	1.29 ± 0.10	1.28 ± 0.10	0.38
HGI	1.06 ± 0.09	1.05 ± 0.09	1.08 ± 0.09	1.10 ± 0.09	0.59
LDL-chol (mmol·L^-1^)					
NGI	3.23 ± 0.19	3.31 ± 0.17	3.39 ± 0.18	3.16 ± 0.14	0.24
HGI^3^	2.85 ± 0.27	3.08 ± 0.29	3.06 ± 0.26	3.13 ± 0.30	0.86
TAG (mmol·L^-1^)^4^					
NGI	1.01 (0.80, 1.27)	1.08 (0.85, 1.36)	1.02 (0.77, 1.35)	1.07 (0.78, 1.49)	0.94
HGI	1.64 (1.16, 2.32)	1.87 (1.30, 2.68)	1.68 (1.23, 2.29)	1.71 (1.25, 2.34)	0.75
Non-HDL-chol (mmol·L^-1^)					
NGI	3.71 ± 0.22	3.84 ± 0.19	3.90 ± 0.22	3.72 ± 0.20	0.11
HGI	3.71 ± 0.30	4.02 ± 0.33	3.88 ± 0.31	3.97 ± 0.33	0.49
Apolipoprotein B (g·L^-1^)					
NGI	0.89 ± 0.06	0.93 ± 0.05	0.94 ± 0.06	0.90 ± 0.05	0.28
HGI	0.94 ± 0.07	0.99 ± 0.07	0.97 ± 0.07	0.97 ± 0.07	0.43
Apolipoprotein AI (g·L^-1^)					
NGI	1.55 ± 0.08	1.56 ± 0.07	1.50 ± 0.06	1.56 ± 0.06	0.63
HGI	1.40 ± 0.06	1.42 ± 0.06	1.41 ± 0.05	1.47 ± 0.06	0.39

^1^Values are mean ± SEM unless otherwise indicated. Abbreviations: chol: cholesterol.

^2^P-value for treatment effect comparison between day 43 values of white and whole grain sourdough breads within the NGI and HGI groups, using repeated measures ANCOVA controlling for participant, treatment period and treatment and including d 1 values as a covariate, followed by the Tukey's test for multiple comparisons.

^3^Within the HGI group, n = 12 for whole grain wheat sourdough bread (Days 1 and 43) and n = 13 for white bread (Days 1 and 43).

^4^Data were log-transformed prior to statistical analysis and presented as geometric mean (95% CI).

The effect of whole grain wheat sourdough bread consumption on serum lipids was significantly influenced by presence of the *APOE *E3/E3 genotype. Within the NGI participants, LDL-cholesterol significantly increased following consumption of whole grain wheat sourdough compared to white bread in participants with the *APOE *E3/E3 genotype (Table 5) but not in participants with the E2/E3 or E3/E4 genotypes (data not shown). Within the HGI participants, TAG and TAG:HDL-cholesterol significantly increased following consumption of whole grain wheat sourdough compared to white bread in participants with the *APOE *E3/E3 genotype (Table 5) but not in participants with the E2/E3 or E3/E4 genotypes (data not shown). There were no significant differences in any other serum lipids or lipid ratios between the whole grain wheat sourdough and white bread treatments in participants with the *APOE *E3/E3 genotype, within either the NGI or HGI group (Table 5). Similarly, there were no significant differences in serum lipids or lipid ratios between the whole grain wheat sourdough and white bread treatments in participants with the *LIPC *-514C>T or LIPC -250G>A genotypes, within either the NGI or HGI group (data not shown).

**Table 5:** Effect of 6-wk whole grain whole wheat sourdough bread consumption on fasting serum lipids in NGI and HGI adults with the APOE E3/E3 genotype^1^

	APOE E3/E3				
	**Whole grain wheat sourdough bread**		**White bread**		**P-value treatment effect^2^**
		
	**Day 1**	**Day 43**	**Day 1**	**Day 43**	

n					
NGI	7	7	7	7	
HGI	9	9	10	10	
Total-chol (mmol·L^-1^)					
NGI	5.02 ± 0.30	5.18 ± 0.29	5.06 ± 0.38	4.93 ± 0.34	0.15
HGI	4.84 ± 0.31	5.34 ± 0.29	5.09 ± 0.28	5.22 ± 0.33	0.15
HDL-chol (mmol·L^-1^)					
NGI	1.26 ± 0.15	1.23 ± 0.13	1.14 ± 0.10	1.14 ± 0.13	0.62
HGI	1.05 ± 0.13	1.05 ± 0.13	1.08 ± 0.13	1.12 ± 0.12	0.49
LDL-chol (mmol·L^-1^)					
NGI	3.22 ± 0.28	3.39 ± 0.29	3.40 ± 0.34	3.14 ± 0.26	0.02
HGI^3^	2.84 ± 0.31	3.20 ± 0.35	3.10 ± 0.26	3.23 ± 0.35	0.45
TAG (mmol·L^-1^)^4^					
NGI	1.10 (0.73, 1.67)	1.17 (0.79, 1.72)	1.07 (0.69, 1.67)	1.24 (0.68, 2.27)	0.40
HGI	1.83 (1.17, 2.86)	2.31 (1.52, 3.51)	1.93 (1.37, 2.73)	1.83 (1.26, 2.66)	0.03
Non-HDL-chol (mmol·L^-1^)					
NGI	3.76 ± 0.35	3.95 ± 0.36	3.92 ± 0.41	3.79 ± 0.38	0.08
HGI	3.79 ± 0.33	4.29 ± 0.35	4.01 ± 0.31	4.10 ± 0.37	0.07
Total-chol:HDL-chol					
NGI	4.30 ± 0.49	4.52 ± 0.49	4.66 ± 0.47	4.63 ± 0.50	0.24
HGI	5.14 ± 0.63	5.76 ± 0.75	5.23 ± 0.59	5.17 ± 0.61	0.10
LDL-chol:HDL-chol					
NGI	2.82 ± 0.40	3.01 ± 0.41	3.15 ± 0.39	2.99 ± 0.39	0.06
HGI^3^	3.01 ± 0.40	3.18 ± 0.49	3.04 ± 0.38	3.03 ± 0.43	0.16
TAG:HDL-chol^4^					
NGI	0.91 (0.48, 1.71)	0.98 (0.56, 1.73)	0.96 (0.53, 1.74)	1.13 (0.52, 2.45)	0.48
HGI	1.85 (0.97, 3.53)	2.33 (1.20, 4.53)	1.89 (1.11, 3.23)	1.72 (0.96, 3.09)	0.04
Non-HDL-chol:HDL-chol					
NGI	3.30 ± 0.49	3.52 ± 0.49	3.66 ± 0.47	3.63 ± 0.50	0.24
HGI	4.14 ± 0.63	4.76 ± 0.75	4.23 ± 0.59	4.17 ± 0.61	0.06
Apolipoprotein B (g·L^-1^)					
NGI	0.92 ± 0.09	0.95 ± 0.09	0.94 ± 0.10	0.91 ± 0.08	0.15
HGI	0.98 ± 0.08	1.06 ± 0.08	1.00 ± 0.07	1.01 ± 0.08	0.15
Apolipoprotein AI (g·L^-1^)					
NGI	1.50 ± 0.06	1.50 ± 0.07	1.41 ± 0.05	1.47 ± 0.09	0.24
HGI	1.40 ± 0.09	1.44 ± 0.08	1.41 ± 0.07	1.47 ± 0.08	0.74
Apolipoprotein B:Apolipoprotein AI					
NGI	0.63 ± 0.08	0.65 ± 0.08	0.67 ± 0.08	0.64 ± 0.07	0.08
HGI	0.74 ± 0.09	0.76 ± 0.08	0.73 ± 0.07	0.72 ± 0.08	0.18

^1^Values are mean ± SEM unless otherwise indicated. Abbreviations: chol: cholesterol.

^2^P-value for treatment effect comparison between day 43 values of white and whole grain sourdough breads within the NGI and HGI groups, using repeated measures ANCOVA controlling for participant, treatment period and treatment and including d 1 values as a covariate, followed by the Tukey's test for multiple comparisons.

^3^Within the HGI group, n = 8 for whole grain wheat sourdough bread (Days 1 and 43) and n = 9 for white bread (Days 1 and 43).

^4^Data were log-transformed prior to statistical analysis and presented as geometric mean (95% CI).

## Discussion

The current study measured serum lipids in response to consumption of whole grain wheat sourdough bread compared to white bread for 6 weeks each, using a randomized crossover design. The study concurrently investigated 2 groups of participants that significantly differed in CVD risk and also considered genetic variation in response to the bread treatments through identification of gene polymorphisms related to CVD risk.

Although body measurements and glycemic endpoints were significantly different between the NGI and HGI groups at study entry, which established their metabolic distinction, they did not significantly differ between treatments, within either group. The lack of change in body measurements (body weight, BMI, body fat, waist circumference) suggests that participants followed their habitual diet and physical activity routines throughout the study to minimize potential confounding effects. Noteworthy is that the lack of change in body weight between treatment periods is consistent with previous similar studies [26-28,32,33]. As expected though, during the study, consumption of the high-fiber whole grain wheat sourdough bread caused an increase in dietary fiber intake within both participant groups relative to the white bread. The whole grain wheat sourdough bread also caused an increase in MUFA intake, only within the NGI group, which is less obviously explained, but given the lack of significant difference in energy or other macronutrients, may be due to variation in food record analysis. Of note, participants were not counselled on the macronutrient distribution of their diet and the results show that their macronutrient intake distribution was comparable to that reported in Canadian nutrition surveys [43].

Fasting serum lipids, apolipoproteins and related ratios were not significantly affected by 6-week consumption of whole grain wheat sourdough bread (12.8-14.9 g dietary fiber/d; Table 1) in comparison to white bread (6.75-8.44 g dietary fiber/d; Table 1), within either the NGI or HGI group. These findings are consistent with a study by Jenkins et al. [32] in which serum total-, LDL- and HDL-cholesterol were not significantly affected by 4-week consumption of 2 high-wheat fiber breads (25 g dietary fiber/d) of varying wheat bran particle size compared to low-wheat fiber bread (6 g dietary fiber/d) in hypercholesterolemic adults, although the same study found that serum TAG were significantly reduced by both high-wheat fiber breads. Other related studies have also found significant lipid changes including reduced total-cholesterol:HDL-cholesterol following 2-week consumption of high-protein wheat flakes (21 g dietary fiber/d) in healthy adults [28], reduced total-cholesterol following 3-week consumption of wheat bran (13 g dietary fiber/d) and whole grain wheat (5.7 g dietary fiber/d) cereal in hypercholesterolemic adults [29] and reduced total- and LDL-cholesterol following 3-week consumption of wholemeal compared to refined wheat food products in healthy adults [26]. The most comparable participant group studied in relation to the current study's HGI participants is that of Sabovic et al. [27] who studied adults with dysmetabolic cardiovascular syndrome (defined by obesity, impaired glucose tolerance, dyslipidemia and arterial hypertension) and found significantly decreased total-, LDL- and HDL-cholesterol following 5-week consumption of wheat fiber supplements (21 g dietary fiber/d). It is possible that if the current study's HGI participants had more metabolic dysregularities like the Sabovic et al. [27] participants, the wheat fiber in the whole grain wheat sourdough bread may have significantly influenced serum lipids. Overall, the inconsistent lipid results among the few whole grain wheat intervention studies completed to date highlights the need for further research.

The gene polymorphisms considered in the current study included 3 of the *APOE *gene (E2, E3 and E4) and 2 of the *LIPC *gene (-514C>T and -250G>A). Genotyping results revealed proportions consistent with the general Caucasian population for all 5 SNPs [34,41,44]. When the influence of having the common *APOE *E3/E3 genotype was considered, results showed that the whole grain wheat sourdough bread significantly increased LDL-cholesterol in NGI participants (n = 7) and significantly increased TAG and TAG:HDL-cholesterol in HGI participants (n = 10). Although no other whole grain wheat intervention study has considered genetic variation, many other studies have examined the influence of *APOE *genotypes, particularly since the *APOE *allele variation can account for approximately 7% of the inter-individual variation in total- and LDL-cholesterol [44]. As reviewed by Ordovas et al. [45], *APOE *genotypes have primarily been studied in relation to dietary interventions of fat [46,47], cholesterol [48,49] and dietary fiber [50,51]. Most related to the current study, oat bran intervention studies of 2- [51] and 4- [50] week durations have found significantly greater reductions in total- and LDL-cholesterol in those with the *APOE *E3/E3 genotype [50] and in those who are *APOE *E2 allele carriers [51], in comparison with wheat bran. The inconsistency of these results with those of the current study and the limited number of related studies confirm the need for continued consideration of nutrigenetics in dietary fiber-related intervention studies.

The finding of increased LDL-cholesterol following consumption of whole grain wheat sourdough bread in the NGI participants with the *APOE *E3/E3 genotype was unexpected and may be explained by a shift in utilization of glucose as a substrate for hepatic acetyl-CoA production. Although it is possible that the glucose sensing transcription factor carbohydrate response element binding protein [52] may have influenced LDL-cholesterol in the *APOE *E3/E3 sample within the NGI group, this hypothesis was not tested in the current study.

The findings of increased TAG and TAG:HDL-cholesterol following consumption of the whole grain wheat sourdough bread in the HGI participants with the *APOE *E3/E3 genotype was also unexpected. Given that *APOE *genotypes are not commonly associated with effects on TAG or HDL-cholesterol, clear explanations for these findings are unconfirmed. Recently, however, Wood et al. [41] demonstrated an interaction between the *APOE *and *LIPC *genes in their effect on plasma TAG in the general population. Therefore, a larger sample size in the current study may have allowed for detection of an interaction between the *APOE *and *LIPC *genes. Nonetheless, the results do suggest a modification in TAG metabolism within the HGI relative to the NGI group that is likely associated with their glycemic and insulinemic dysfunction.

The current study has both limitations and strengths that deserve mention. Although the consideration of genetic variation strengthened the current study, there was a limited ability to detect genotype-treatment interactions due to under-representation of the rare *APOE *and *LIPC *polymorphisms. This under-representation was in part due to retrospective genotyping, which although common due to the financial and labour constraints, causes further reason to interpret the results with caution. Also worth noting as a limitation is that particle size was not measured for either of the treatment breads. Strengths of the study include its randomized crossover design, and inclusion of not only the polymorphisms, but 2 participant groups of distinct CVD risk. Also, the study participants maintained their usual diet and since they were provided with the treatment bread, they were not required to self-select their whole grain products, which may help to minimize potential confounding variables. Furthermore the direct comparison of whole grain wheat to refined wheat bread adds clarity to the wheat literature in ascertaining the difference in health effects between whole grain and refined foods.

## Conclusions

In summary, this study uniquely compared whole grain wheat to refined wheat bread and considered the influence of individual CVD risk and genetic variation on serum lipid response. Participants were not counselled on the macronutrient distribution of their diet and their intake was comparable to that reported in Canadian nutrition surveys [43]. Results showed that 6-week consumption of whole grain wheat sourdough bread did not significantly affect serum lipids in comparison to white bread, in adults of either a low or high level of CVD risk. When genetic variation was considered, albeit limited by retrospective assessment, it was found that in those with the *APOE *E3/E3 genotype, whole grain wheat sourdough bread unfavourably increased LDL-cholesterol in NGI participants, and TAG and TAG:HDL-cholesterol in HGI participants, when compared to white bread. These data add to the limited literature of intervention studies examining whole grain wheat relative to refined wheat breads and emphasize the need to consider genetic variation in relation to lipoprotein lipid content and CVD risk.

## List of abbreviations

**ANCOVA**: analysis of covariance; **CFG**: Canada's Food Guide; **CVD**: cardiovascular disease; **g·L^-1^**: grams per liter; **LIPC**: hepatic lipase gene promoter; **HOMA-IR**: homeostasis model assessment of insulin resistance; **HGI**: hyperglycemic/hyperinsulinemic; **mmol·L^-1^**: millimoles per liter; **ng·L^-1^**: nanograms per liter; **NGI**: normoglycemic/normoinsulinemic; **pmol·L^-1^**: picomoles per liter; **SNP**: single-nucleotide polymorphism.

## Competing interests

The authors declare that they have no competing interests.

## Authors' contributions

AJT co-coordinated the participant recruitment and data collection, worked on the sample and statistical analysis, summarized the results and worked on the manuscript preparation. KAM co-coordinated the participant recruitment and data collection, worked on the sample analysis and contributed to the manuscript preparation. LER contributed to the study design, supervised the subject recruitment, data collection and sample analysis and contributed to the manuscript preparation. MB provided expertise for the nutrigenetic analysis and results interpretation and contributed to the manuscript preparation. TEG designed the study, secured the funding, supervised the subject recruitment and data collection and contributed to the manuscript preparation. AMD contributed to the study design, supervised the subject recruitment and data collection, directed the data and statistical analyses, directed the results interpretation and directed the manuscript preparation. All authors read and approved the final manuscript.
